# The Role of the Bactericidal Mechanism of Copper Elements and Its Effect on the Corrosion Resistance of Steel

**DOI:** 10.3390/ma17235921

**Published:** 2024-12-03

**Authors:** Yunsheng Xue, Cheng Ding, Li Gong, Yingxue Teng, Jing Guo, Shanshan Chen

**Affiliations:** 1 School of Materials and Metallurgy, University of Science and Technology Liaoning, Anshan 114051, China; 2 Shi-Changxu Innovation Center for Advanced Materials, Institute of Metal Research, Chinese Academy of Sciences, Shenyang 110016, China; 3 Key Laboratory for Advanced Materials of Ministry of Education, School of Materials Science and Engineering, Tsinghua University, Beijing 100084, China

**Keywords:** copper-containing steel, bactericidal property, corrosion resistance, corrosion mechanism

## Abstract

In this paper, two kinds of copper-containing steels with copper contents of 2.31 and 6.01 wt.% were designed. By comparing with commercial Q355, the bactericidal properties of copper in seawater containing sulfate-reducing bacteria (SRB) and its influence on the corrosion process of steel were revealed. The corrosion rate, morphology of products, and bactericidal action of copper were tracked by scanning electron microscopy, X-ray diffraction, confocal microscopy, and electrochemical analysis techniques. It was found that the resistance of copper-containing steel to bacterial corrosion was obviously better than that of non-copper-containing steel. At 28 days, the weight loss rates in the SRB environment for 0Ni2Cu6 samples increased by merely 5.43%, which was nearly half that of Q355 of 9.75%. Cu-containing steels exhibited potent antibacterial action, with the ε-Cu phase altering the corrosion byproduct composition from brittle flakes to robust particles and inhibiting the production of H_2_S. The killed bacteria adhered to the surface of the steel and slowed down the corrosion of the steel. The confocal laser scanning microscope and electrochemical experiments showed that a dense CuFeO_4_ film formed on the substrate, impeding corrosive ion penetration, and an upsurge in Cu content markedly enhanced the material’s anti-corrosion and antimicrobial attributes.

## 1. Introduction

The rapid development of fields such as shipbuilding, ocean platforms, marine engineering, and offshore oil exploitation has considerably driven the quality advancement of marine-grade steel. The contemporary needs of marine steel not only require higher strength, toughness, and good weldability, but also outstanding resistance to seawater and microbial corrosion.

The marine environment is complex and ever-changing [1]. In addition to the corrosion caused by chloride ions, pH [2], and other salts in seawater, the biofilms formed by microbes on metal surfaces also affect metal corrosion [3], with sulfate-reducing bacteria being the main corrosive bacteria [4,5]. Alabbas [6] and Castaneda [7] have found that the formation of biofilms can have both positive and negative feedback effects on microbial corrosion. This is due to the involvement of different organisms in the corrosion process. These microbes form biofilms on the steel substrate and act in consortia, adhering together to exert their effects. For example, Liu’s research indicates that the combined action of sulfate-reducing bacteria and sulfur-oxidizing bacteria (SOB) can lead to more severe corrosion of 2205 duplex stainless steel [8]. Similarly, San’s study found that biofilms of Pseudomonas aeruginosa have a negative feedback effect on the corrosion of alloy steel, impeding the corrosion of the steel substrate [9]. Therefore, developing a novel steel that is both corrosion-resistant and antibacterial has become increasingly important.

The addition of copper into steel grades can take into account the high strength and toughness, corrosion resistance, low temperature impact toughness, and good machinability and weldability of steel [10]. Another advantage of copper is its corrosion resistance, which is the key to ensuring the service life of steel in a marine environment with high salt and high humidity [11,12]. The addition of Cu into steel can facilitate accurate microstructure tailoring and improved strength and hardness indexes via the designed thermal treatment process [10,13,14,15].

Moreover, the corrosion resistance of steel is vastly enhanced, and the precipitated ε-Cu [16] phase in steel also exhibits strong antibacterial performance [17,18]. A large number of studies on the antibacterial activity and corrosion of copper-containing steel have shown [19,20,21] that copper-containing steel has the dual characteristics of both structural material and antibacterial function. Therefore, it is of importance in terms of application and theoretical value to study and discuss the microbial corrosion resistance of copper-containing steel.

Due to the controversies between various corrosion mechanisms, further research into the antibacterial mechanism of copper-containing steel is imperative. To this end, we designed two types of copper-containing steel with different copper contents. Using SRB as the pathogenic bacteria, we simulated the corrosion and antibacterial behavior of Cu-containing steel in a marine environment, aiming to explore the impact of Cu content on the corrosion resistance and antibacterial properties of Cu-containing steel. This research provides a theoretical basis and experimental data for the development of high-strength, Cu-bearing steel.

## 2. Materials and Methods

### 2.1. Experiment Materials

**Figure 1:**
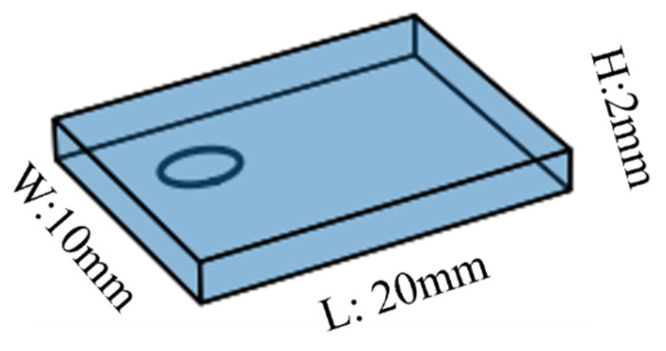
Sample shape.

Three types of sample steels were used: Q355, 0Ni2Cu2, and 0Ni2Cu6. Their compositions are presented in Table 1, and their shapes are illustrated in Figure 1. Early studies have shown that the antibacterial effect of Cu-steel can be improved when Cu elements are precipitated in the ε-Cu phase [17]. Appropriate heating and cooling treatment of Cu-containing steel is required to ensure uniform precipitation of the ε-Cu phase. The samples were heated at 950 °C for 5 min, then water-cooled to room temperature, and then reheated at 850 °C for 12 h and air-cooled to room temperature. The samples were sanded with 2000-grit sandpaper, soaked in ethanol for one hour, and then sterilized under UV light for one hour to be completely sterile before testing.

The seawater was artificially configured, and its main component configuration is shown in Table 2. Before the experiment, the seawater should be injected with nitrogen to eliminate oxygen in the solution, and then placed in a high-temperature sterilization kettle with a temperature of 121 °C for two hours.

### 2.2. SRB Bacteria Culture

SRB was extracted from Dalian marine clay and enriched in a Postgate C medium, and its chemical composition is shown in Table 3. Before starting the experiment, the medium was autoclaved at 121 kPa, 120 °C for 30 min and continuously ventilated with sterile nitrogen for 1 h to evacuate the air from the medium. The pH value of the medium was maintained at 8.0–8.2, and regular nutrient supplementation was ensured during the experiment. The concentration of sulfate-reducing bacteria was measured by applying a hemocytometer with an initial cell concentration of 10^6^ cells/mL. In the process of the experiment, the medium was injected into the experimental device regularly and quantitatively to avoid experimental errors caused by the massive death of sulfate-reducing bacteria. The steel samples were soaked in alcohol for 5 min, then blow-dried and immediately put into the solution for the soaking experiment.

### 2.3. Experiment Methods

Each kind of experiment consisted of three parallel samples all hung with nylon lines in sterile seawater and SRB seawater for 28 d, respectively, maintaining constant temperature and humidity. At the end of immersion, the samples were rinsed twice with saline clear rinse and 2.5% pentanediol for 4 h, and then washed with distilled water. Dehydration was carried out for 10 min using different concentrations of alcohol, followed by freeze-drying.

The surface morphology was observed by scanning electron microscopy (Sigma 500, ZEISS, Steinheim, Germany). The type of detector for the imaging was secondary electrons, and the chemical contents of the corrosion product were examined by EDS. The corrosion crater phase was observed by XRD (X’Pert Powder, Malvern Panalytical, Almelo, The Netherland). The state of the bacteria on the surface of the sample was observed using a confocal laser scanning microscope (CLSM, C2 Plus, Nikon, Tokyo, Japan). The wavelengths stimulated by the shooting were 488 nm and 559 nm, the magnification was 200 times, and the 5 fields of view were shot at the edge and the center with a 2 mm interval.

The samples were immersed in 500 mL of treated medium, and purified and proliferated sulfate-reducing bacteria were taken into the container and sealed for a 14-day sulfate-reducing bacteria fouling resistance test. The samples were removed and gently rinsed with a phosphate-buffered saline (PBS) solution to remove the floating cells. Staining was performed with SYTO-9 (fluorescent green) and propidium iodide (fluorescent red) (Invitrogen, Eugene, OR, USA). The cells were imaged by confocal microscopy for dead or alive staining, with green dots at an excitation wavelength of 488 nm for live cells and red dots for dead cells at an excitation wavelength of 559 nm.

Electrochemical tests were conducted using a Vertex Ctype electrochemical workstation (CHI600F, CH Instruments, Austin, TX, USA). The exposed area was 1 cm^2^, and the rest was sealed with epoxy resin and subjected to electrochemical experiments by immersion for 3, 7, 14, and 28 days; three sets of parallel experiments were set up for each test. A saturated calomel electrode was used as the reference electrode, and a platinum sheet electrode was used as the auxiliary electrode. The sample to be tested was used as the working electrode. The open circuit potential (OCP) was tested for 120 min. The potential scanning range was −2–2 V, the scanning rate was 0.01 V/s, and the impedance frequency range was 10^−2^–10^5^ Hz. The substitute circuit was fitted with ZView software (V 2.70). The corresponding electrochemical parameters of corrosion current density (Icorr, A cm^−2^) and corrosion potential (Ecorr, V) were measured from the polarization curves using ZsimpWin software (V 3.60).

## 3. Result

### 3.1. Weight Loss During Immersion

**Figure 2:**
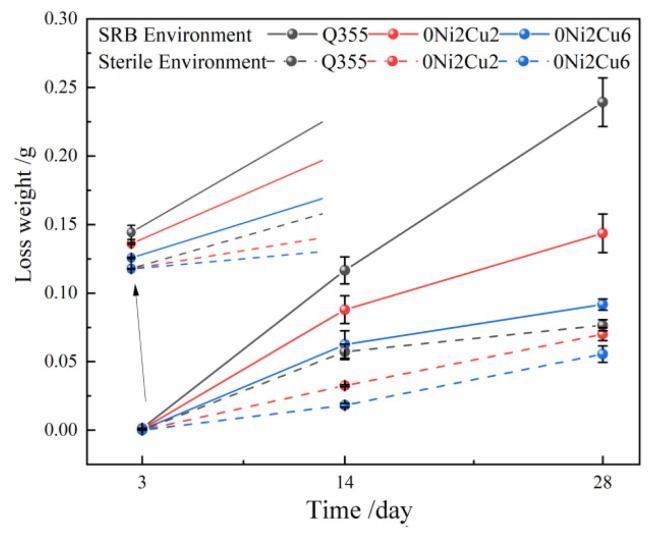
Weight loss curves of experimental steel in different environments.

Figure 2 presents the weight loss curves of three steels soaked in sterile and SRB seawater for 3, 14, and 28 days. As corrosion progressed, the weight loss in the presence of bacteria was consistently greater than that in a sterile environment, with all three types of steel showing an increasing trend in weight loss. Additionally, the corrosion rate (slope of the curves) changed with corrosion time. Comparing the corrosion weight loss curves of the three steels, it was obvious that the loss rate of the two Cu-containing steels was significantly lower than that of the Cu-free Q355 samples. Furthermore, the higher the Cu content, the lower the weight loss. Specifically, at 28 days, the weight loss rates in the SRB environment for Q355, 0Ni2Cu2, and 0Ni2Cu6 experimental steels increased by 9.75%, 6.64%, and 5.43% respectively, compared to those in the sterile environment. This indicates that an increase in Cu content could effectively inhibit the accelerated corrosion by bacteria, with a clear trend that higher Cu content yielded more significant inhibition effects.

### 3.2. Corrosion Morphology and Analysis of Corrosion Products

#### 3.2.1. Analysis of Corrosion Morphology

**Figure 3:**
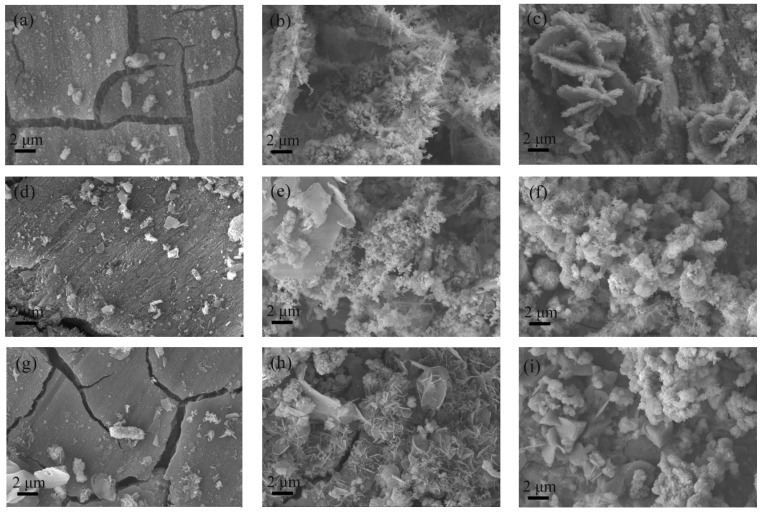
Corrosion morphology of SRB in different samples: Q355: (**a**) 3 days, (**b**) 14 days, (**c**) 28 days; 0Ni2Cu2: (**d**) 3 days, (**e**) 14 days, (**f**) 28 days; 0Ni2Cu6: (**g**) 3 days, (**h**) 14 days, (**i**) 28 days.

Figure 3 shows the corrosion morphology of different samples after immersion for various durations. From the SEM photos, it is evident that there were minimal corrosion products at 3 days, with isolated bacteria observed attached to the steel substrate. The cracks on the sample surface were due to the dehydration of the recently formed thin corrosion layer. At the onset of corrosion, when the sample was dried, hydrate Fe(OH)_3_ in the corrosion products lost its crystallization water, leading to volume shrinking and consequent cracking. With ongoing corrosion, by 14 days, there was a visible increase in corrosion products, which had formed FeOOH crystals that encapsulated the bacteria adhering to the steel substrate. The surface of the Cu-free Q355 sample was heavily corroded, with a thick layer of corrosion products completely covering it. In contrast, the steel substrate was still visible on the Cu-bearing steel surfaces after 14 days, with 0Ni2Cu6 steel showing the fewest corrosion products. Regarding the morphologies, the corrosion products were mostly needle-like in Q355; spherical and granular in 0Ni2Cu2; and of rose-petal appearance in 0Ni2Cu6. As corrosion continued, after 28 days, the surfaces of all three types of steel samples were completely covered with corrosion products. Among them, a massive attachment of bacteria on the corrosion products and a biofilm were found only on the surface of the Cu-free Q355. In Q355 samples, the volume of corrosion products significantly decreased at 28 days compared to that of 14 days, possibly due to the severe corrosion and partial detachment of the rust. At 28 days, the corrosion product morphology of the two Cu-containing steels was consistent, exhibiting spherical external rust with needle-like corrosion products attached, while the inner rust remained predominantly petal-shaped. In terms of the quantity of corrosion products, 0Ni2Cu6 had the fewest. Therefore, it was evident that the corrosion products of Cu-free Q355 steel were the most abundant, and its corrosion rate was the fastest, while the corrosion products of 6.04% Cu-bearing steel were the fewest, and its corrosion rate was the slowest.

**Figure 4:**
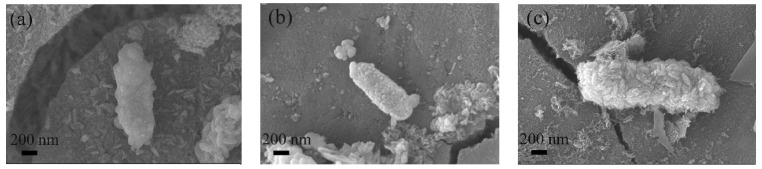
Bacterial morphology of SRB in different samples at 3 days: (**a**) Q355; (**b**) 0Ni2Cu2; (**c**) 0Ni2Cu6.

To understand the adhesion and growth mechanism of bacteria on the steel surface, the samples soaked for three days were observed at high magnification. The selected duration for three-day soaking was contingent upon the fact that the surface of the samples did not yet exhibit a substantial quantity of corrosion products to encapsulate the bacteria, making it easier to observe the morphology of the bacteria. Figure 4 shows the morphology of SRB after three days of soaking. It reveals that the bacterial morphology on Q355 steel was full and smooth, presenting the characteristic features of bacterial viability. In contrast, when examining the bacterial surfaces on 0Ni2Cu2 and 0Ni2Cu6, they were covered with a layer of iron oxide filiform corrosion products, which could easily lead to speculations that the bacteria attached to the Cu-containing steel surfaces of 0Ni2Cu2 and 0Ni2Cu6 may have already died, allowing the corrosion products to crystallize on their outer surfaces.

#### 3.2.2. Analysis of Corrosion Products

**Figure 5:**
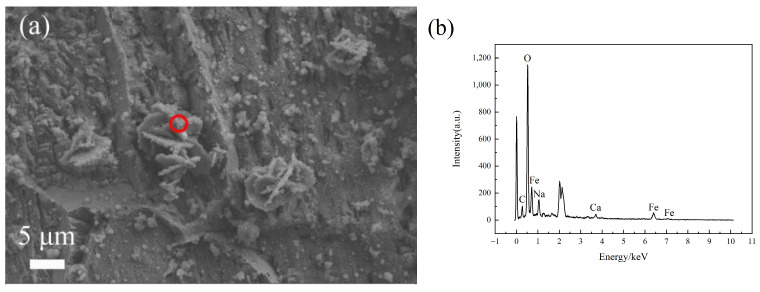

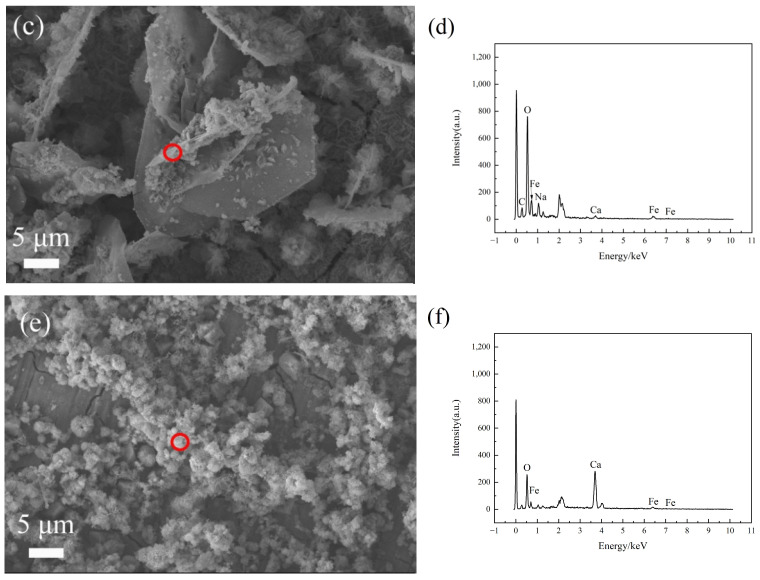
Morphology of corrosion products and their elemental distribution for three samples immersed in an SRB environment for 28 days. (**a**,**c**,**e**) Corrosion morphology and (**b**,**d**,**f**) the relevant chemical contents of corrosion products examined by EDS for Q355 (**a**,**b**), 0Ni2Cu2 (**b**,**d**), and 0Ni2Cu6 (**e**,**f**), the EDS scanning position is marked with a red circle.

Figure 5 presents the observations of the corrosion products from three different steel samples, revealing distinct morphologies. The corrosion layer on Q355 appeared as small spheroidal shapes intermixed with rust layers exhibiting a cruciform pattern on the surface, along with even finer particles. Energy-dispersive spectroscopy (EDS) analysis showed that the corrosion products were primarily composed of Fe and O. Within the corrosion products of the 0Ni2Cu2 sample, large petal-like layers were observed, with bacteria visible on the surface of these flaky rust layers, and EDS analysis also indicated a composition mainly composed of Fe and O. The corrosion morphology of the 0Ni2Cu6 sample exhibited a granular appearance, and besides Fe and O, elements of Cu were also detected in the EDS of its corrosion products.

**Figure 6:**
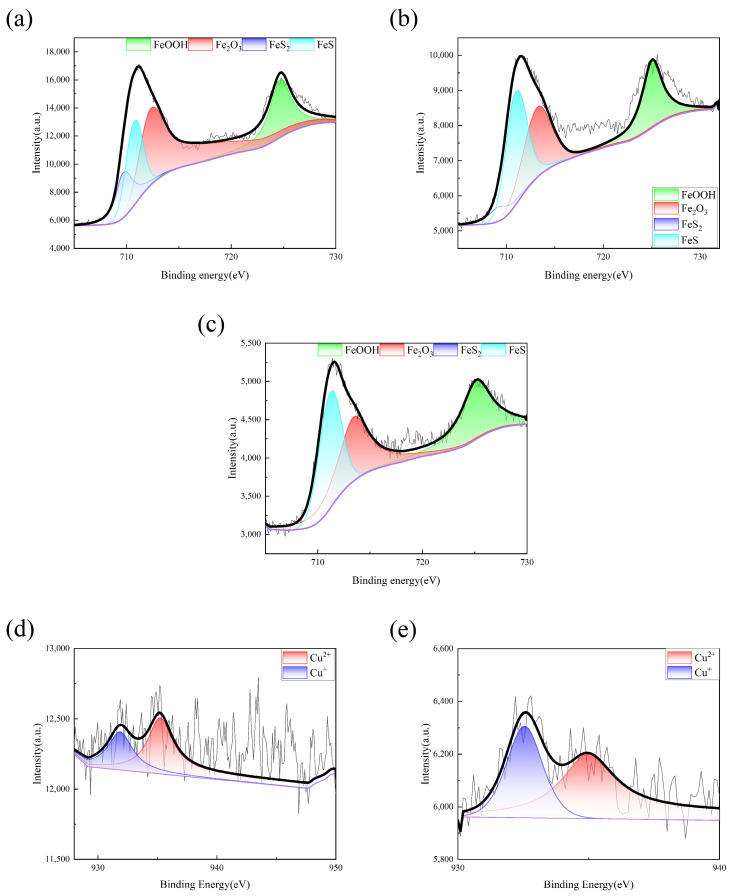
XPS results of corrosion products of three specimens corroded for 28 days: (**a**) Q355 Fe2p; (**b**) 0Ni2Cu2 Fe2p; (**c**) 0Ni2Cu2 Fe2p; (**d**) 0Ni2Cu2 Cu2p; and (**e**) 0Ni2Cu6 Cu2p.

An X-ray photoelectron spectroscopy (XPS) analysis was conducted to further confirm the composition of Fe_2p_ and Cu_2p_ within the corrosion products, as shown in Figure 6 and Table 4. The Fe_2p_ and Cu_2p_ XPS spectra and atomic concentrations for the three samples within an SRB environment showed a hierarchy in the quantity of corrosion products. Specifically, the two types of Cu-containing steels exhibited an equivalent quantity of these products, which were dominated by FeS, aligning as FeS > Fe_2_O_3_ > FeOOH. However, in the Cu-free steel, the most plentiful corrosion product was Fe_2_O_3_, with the sequence of Fe_2_O_3_ > FeS > FeOOH > FeS_2_. It is noteworthy that FeS_2_ was exclusively identified in Cu-free steels and not in Cu-containing ones. This finding suggests for potential future research to investigate the potential role of Cu in facilitating the conversion of FeS_2_ to FeS.

According to the Cu_2p_ XPS results and the analysis derived from the graphs, it was discernible that the Cu valence states in 0Ni2Cu2 and 0Ni2Cu6 steels were consistently Cu^+^ < Cu^2+^. Concurrently, the precipitated Cu^+^ and Cu^2+^ ions can affect the lattice structure of FeOOH, promoting the nucleation and crystallization of α-FeOOH [19]. The presence of α-FeOOH contributes to a denser and more stable rust layer that effectively impedes corrosive ions from contacting the steel substrate, thereby mitigating corrosion.

### 3.3. Laser Confocal Analysis

To further analyze the antibacterial performance of Cu-bearing and Cu-free carbon steels, laser confocal microscopy experiments were conducted to offer a detailed comparison assessment of the germicidal properties of carbon steels comprising different Cu contents.

**Figure 7:**
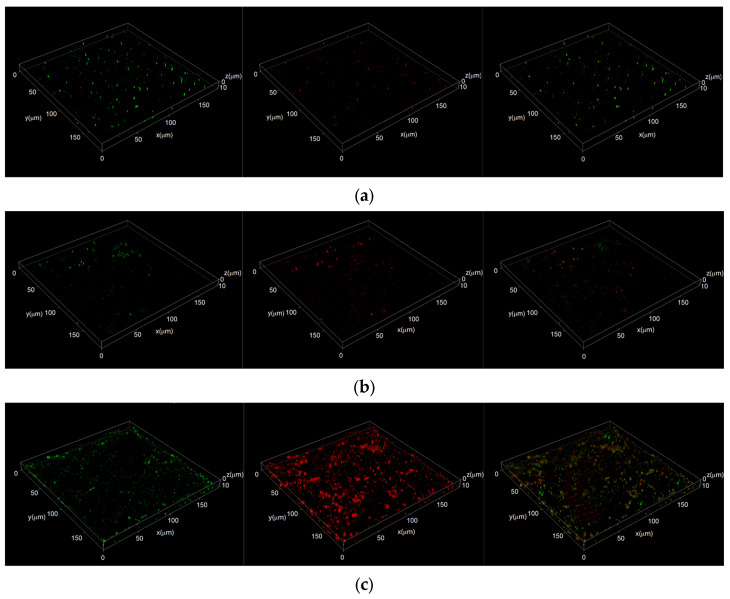
Laser confocal results of different samples after 14 days of immersion: (**a**) Q355; (**b**) 0Ni2Cu2; (**c**) 0Ni2Cu6: green fluorescence: live bacteria, red fluorescence: apoptotic bacteria.

Figure 7 presents the results of laser confocal microscopy after a 14-day immersion. SYTO-9 was used to stain live and dead bacteria, with green fluorescence representing live bacteria and red fluorescence indicating apoptotic bacteria. The tertiary image represents a composite of the aforementioned fluorescence. These findings reveal that the surface of the Q355 steel was covered with a large number of bacteria, as evidenced by the dominant green fluorescence as opposed to the less pronounced red fluorescence. A small number of bacterial deaths as seen via the apoptosis indicator is a reflection of metabolic activity during the bacterial life cycle. Therefore, it was deduced that the Q355 steel surface did not substantially deter bacterial survival, thereby sustaining an environment conducive to bacterial proliferation.

In contrast, the Cu-bearing steels, specifically 0Ni2Cu2 and 0Ni2Cu6, displayed greater areas of red fluorescence than green fluorescence, suggesting a high incidence of bacterial apoptosis, which thus confirmed the antibacterial effect of the Cu content in the steel. Moreover, the area of red fluorescence on the 0Ni2Cu6 samples was larger than that on the 0Ni2Cu2 samples, indicating a more robust antibacterial performance of the 0Ni2Cu6 sample.

The germicidal capability of the 0Ni2Cu2 and 0Ni2Cu6 steels was primarily attributed to the ε-Cu precipitate phase formed in the heat-treated copper-containing steels. The Q355 steel without Cu did not exhibit any discernible antibacterial effect on SRB. This finding supports prior observations that bacteria on the surfaces of Cu-bearing steel samples, i.e., 0Ni2Cu2 and 0Ni2Cu6, may have already perished post-immersion. Considering the differences in Cu content, it was noted that the steel with 6.01% of Cu (0Ni2Cu6) exhibited superior antibacterial properties compared to those of the steel with 2.31% of Cu (0Ni2Cu2).

### 3.4. Surface Morphology and Phase Analysis of the Substrate’s Corrosion Pits

**Figure 8:**
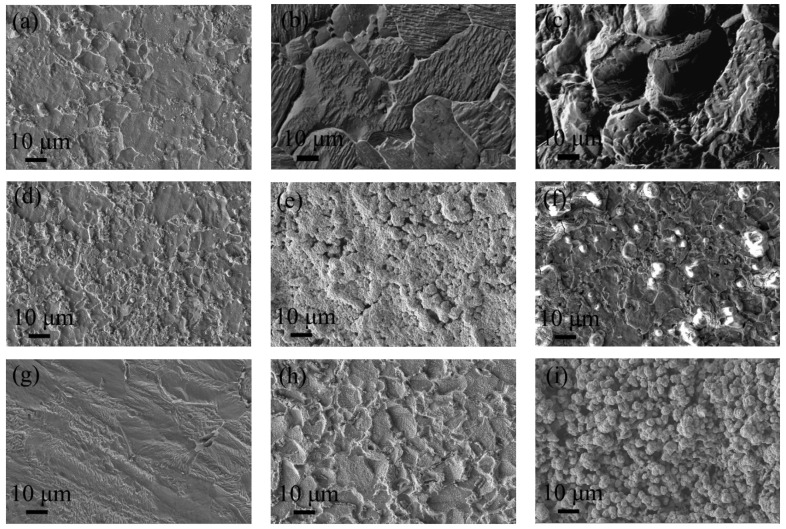
Corrosion pit shape of different experimental steels at different stages of immersion: Q355 (**a**) 3 days; (**b**) 14 days; (**c**) 28 days; 0Ni2Cu2 (**d**) 3 days; (**e**) 14 days; (**f**) 28 days; 0Ni2Cu6 (**g**) 3 days; (**h**) 14 days; (**i**) 28 days.

Figure 8 depicts the morphologies of corrosion pits forming on different experimental steels over various durations of immersion. Examination of the three experimental steels at different stages of pitting revealed that Q355 suffered the most severe corrosion after 3 days of immersion, displaying smaller pitting pits indicative of a lower corrosion degree. This condition exacerbated after 14 days, marked by intergranular corrosion, and the pits had further enlarged and deepened after 28 days. The steel 0Ni2Cu6 exhibited the minimal degree of corrosion, with almost no signs of corrosion after 3 days, deepened pits after 14 days, and granular protrusions after 28 days. The corrosion degree of 0Ni2Cu2 occupied a middle ground between the other two steels, developing spherical granular protrusions within 14 days that had vanished by the 28th day, leaving behind a surface scattered with shallow and finer pits. Generally, the corrosion patterns observed on Q355, 0Ni2Cu2, and 0Ni2Cu6 were distinct, with Q355 primarily undergoing surface corrosion post SRB exposure, and the two types of Cu-containing steels mostly experiencing pitting corrosion. The hierarchy of corrosion severity was Q355 > 0Ni2Cu2 > 0Ni2Cu6.

**Figure 9:**
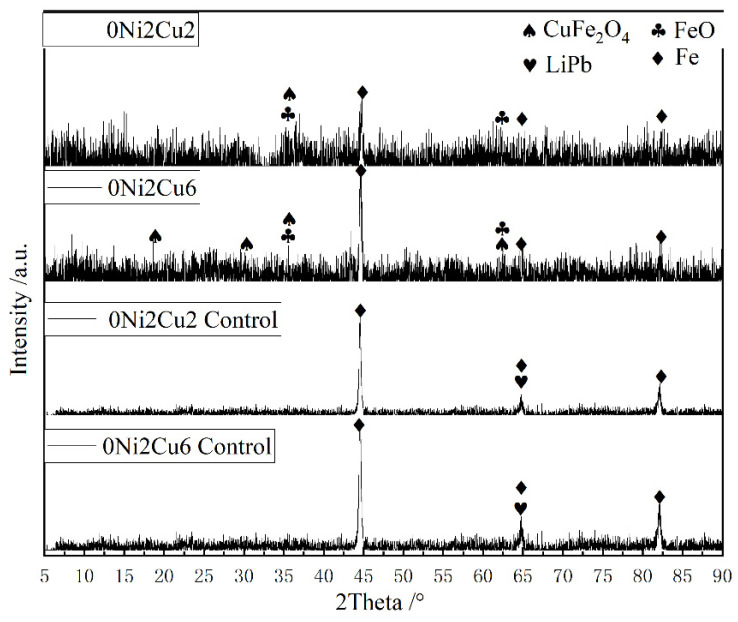
XRD plots of etch pits of two Cu-containing steels immersed in different SRB environments for 28 days.

To discern the composition of the acid-resistant granular protrusions on the surfaces of the 0Ni2Cu2 and 0Ni2Cu6 samples, an XRD phase analysis was performed before and after a 28-day immersion followed by acid pickling, as shown in Figure 9. The initial phase analysis of the as-received samples only revealed diffraction peaks of iron for 0Ni2Cu2 and 0Ni2Cu6. After immersion, diffraction results of these samples revealed numerous additional peaks, including the characteristic peak of CuFeO_4_, implicating this compound as a probable primary constituent of the granular protrusions persisting on the surfaces after acid pickling.

### 3.5. Electrochemical Measurement Analysis

**Figure 10:**
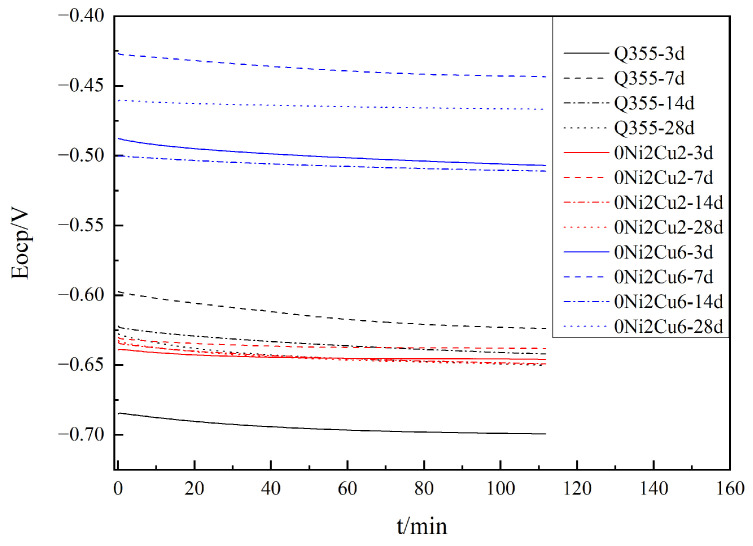
OCP diagrams of different samples immersed in SRB for different numbers of days.

Figure 10 Shows the OCP diagrams of different samples immersed in SRB. As can be seen, the OCP of Q355 was the lowest when it was soaked for 3 days, that of 0Ni2Cu2 was high, and that of 0Ni2Cu2 was the highest. Among them, the OCP of 0Ni2Cu2 was much higher than that of the other two samples at all times. That means the surface of 0Ni2Cu2 was relatively stable.

**Figure 11:**
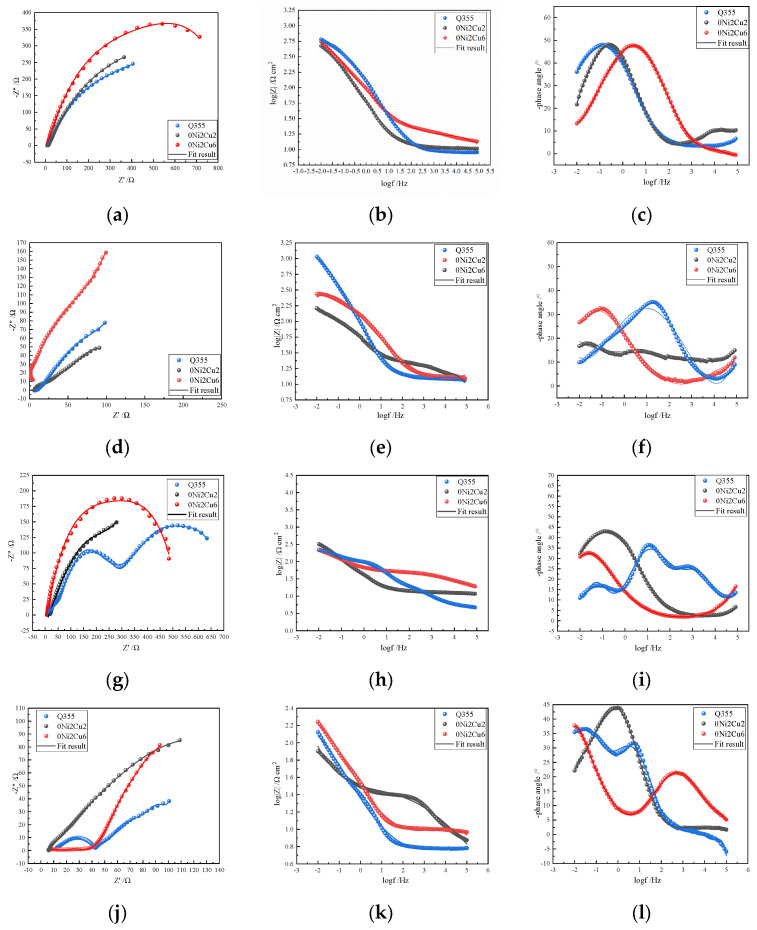
Electrochemical analyses of different samples immersed in SRB for different numbers of days: immersed for 3 days: (**a**) Nyquist plot and (**b**,**c**) Bode plots; immersed for 7 days: (**d**) Nyquist and (**e**,**f**) Bode plots; immersed for 14 days: (**g**) Nyquist plot and (**h**,**i**) Bode plots; immersed for 28 days: (**j**) Nyquist plot and (**k**,**l**) Bode plots.

Figure 11 presents Nyquist and Bode plots at different immersion intervals, with electrochemical curves for the different samples clearly varying under identical environmental conditions. It is known that bacteria accumulate over time on steel substrates, forming biofilms that hinder the contact between corrosive ions and the substrate. Thus, the size of the impedance arc in Nyquist plots changed with the growth of the biofilm and corrosion duration.

Nyquist plots collected at incremental times illustrate that during the initial stages of corrosion (3 days), the impedance arc of the Cu-doping steel was greater than that of the Cu-free carbon steel, demonstrating its superior corrosion resistance from the outset. Advancing into the corrosion timeline (7 days), Q355 exhibited augmented resistance, primarily due to the formation of a more substantial rust layer that served as a barrier against the infiltration of corrosive ions, culminating in an impedance arc radius that overshadowed that of 0Ni2Cu2. Approaching the mid-stage of corrosion (14 days), Q355’s impedance arc dramatically decreased, possibly due to the detachment of an overly thick biofilm or corrosion product layer, allowing corrosive ions to directly contact the steel substrate surface, hence reducing corrosion resistance. Meanwhile, 0Ni2Cu6 maintained higher corrosion resistance than 0Ni2Cu2 did. In the latter stages of corrosion (28 days), 0Ni2Cu6 had the largest impedance arc and Q355 the smallest; 0Ni2Cu6 continued to exhibit superior corrosion resistance.

**Figure 12:**
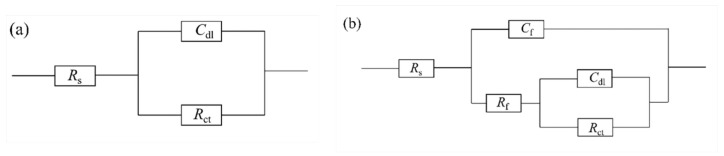
Fitted circuit diagrams of SRB immersed in different samples for different days: (**a**) immersed for 3 days; (**b**) immersed for 7, 14, and 28 days.

Figure 12 presents the fitted circuit diagrams, with (a) representing the equivalent circuit before biofilm formation at three days and (b) representing the equivalent circuit during the middle and later stages of corrosion: R_s_ stands for the solution resistance, C_dl_ represents the double-layer capacitance, and R_ct_ represents the charge transfer resistance. Due to the formation of rust layers and bacterial biofilms in the middle and later stages, an equivalent circuit (b) incorporates an additional rust layer, biofilm resistance (R_f_), and biofilm capacitance (C_f_). The impedance fitting data are shown in Table 5. The highest resistance value occurred on 0Ni2Cu6 steel after 28 days of immersion, a time at which its surface had a layer of granular protrusions that effectively hindered corrosive ions from penetrating into the steel substrate and causing corrosion. By combining the Nyquist plots and fitting data, it became clear that overall, due to the presence of Cu, copper-containing steels displayed superior corrosion resistance compared to that of Cu-free carbon steels, and as corrosion progressed, the corrosion resistance of Cu-bearing steels intensified, with the corrosion performance of steel containing 6.01% of Cu outperforming that of 2.31% Cu content.

**Figure 13:**
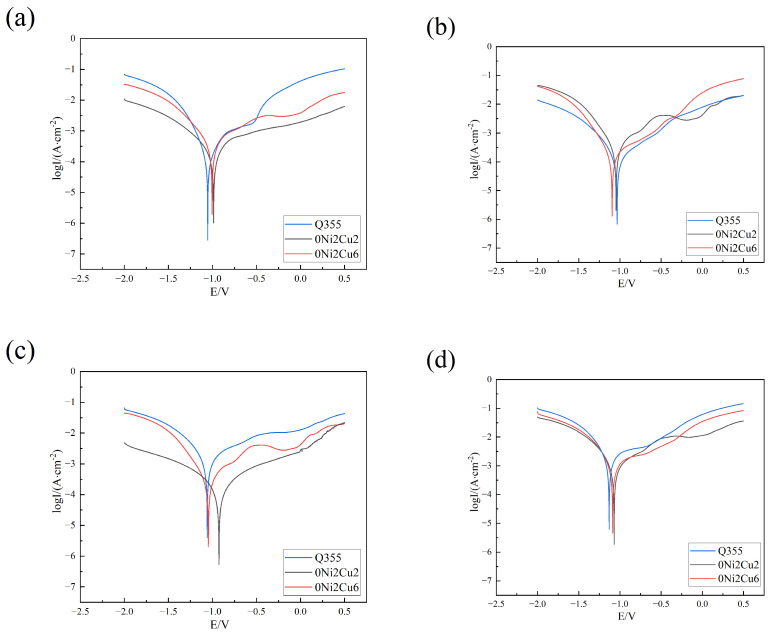
Polarization curves of different samples immersed in SRB for different numbers of days: (**a**) 3 days; (**b**) 7 days; (**c**) 14 days; and (**d**) 28 days.

Figure 13 and Table 6 present the electrochemical Tafel polarization curves and their fitting data. After 3 days of corrosion, 0Ni2Cu6 exhibited excellent corrosion resistance with I_corr_ = 1.648 × 10^−5^, while Q355 displayed the worst one with I_corr_ = 3.757 × 10^−4^. As corrosion continued, after 7 days of immersion, 0Ni2Cu6 maintained the best corrosion resistance with I_corr_ = 1.083 × 10^−4^. After 14 days, the self-corrosion current densities of 0Ni2Cu2 and 0Ni2Cu6 were similar with I_corr_ = 2.867 × 10^−4^ and 2.514 × 10^−4^, respectively, and Q355 had the highest with I_corr_ = 7.838 × 10^−4^. Upon reaching 28 days, the self-corrosion current density of Q355 differed by an order of magnitude from that of the Cu-containing samples with I_corr_ = 5.556, 4.584 × 10^−4^, and 3.905 × 10^−4^ for Q335, 0Ni2Cu2, and 0Ni2Cu6, respectively, indicating that Cu-bearing steels exhibited substantially superior corrosion resistance during this stage. The polarization results were consistent with the impedance findings.

### 3.6. Discussion

The differences observed in these results can be substantially ascribed to Cu content, as varying levels of Cu significantly affect bactericidal efficacy and enhance corrosion resistance. A schematic illustration for a corrosion mechanism is summarized in Figure 13 for a better understanding. Early research found that Cu atoms precipitate as ε-Cu on the steel substrate surface after heat treatment, leading to the formation of a protective oxide layer on the surface [12]. It has been reported that when Cu is added into steels, Cu ions are produced during corrosion, which can then form an oxide layer on the surface of the Cu-doping steels. The involved reaction equations are shown in Equations (1) to (5):

(1) $$4\mathrm{Cu}+O_{2}+2H_{2}O\to 4{\mathrm{Cu}}^{+}+4{\mathrm{OH}}^{-}$$

(2) $${\mathrm{Cu}}^{+}+{\mathrm{Cl}}_{2}\to {{[\mathrm{CuCl}}_{2}]}^{-}$$

(3) $${\mathrm{Cu}}^{+}\to {\mathrm{Cu}}^{2+}+\mathrm{Cu}$$

(4) $${\mathrm{Cu}}^{2+}+{\mathrm{Fe}}_{2}O_{4}^{2-}\to \mathrm{Cu}{\mathrm{Fe}}_{2}O_{4}$$

(5) $${\mathrm{Cu}}^{2+}+2{\mathrm{OH}}^{-}\to \mathrm{Cu}{\left(\mathrm{OH}\right)}_{2}$$

**Figure 14:**
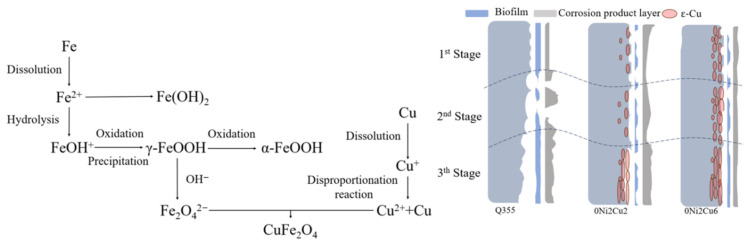
Corrosion mechanism diagram.

Figure 14 illustrates the corrosion mechanism intended to facilitate a comprehensive understanding. It reveals that the fluctuation in Cu content, which significantly impacts the physiological state of SRB, the diversity of the biofilms, and the presence of Cu-rich granular protrusions, is the primary factor dictating the corrosion results. The corrosion process can be divided into three stages. In the first stage, Q355, lacking bactericidal properties, allows SRBs to thrive and produce biofilms. During the second stage, these biofilms, along with corrosive ions, accelerate the corrosion of the steel substrate, resulting in a large amount of loose corrosion products that detach due to gravity along with the biofilms. This causes the Q355 substrate to be exposed to the corrosive medium in the last stage, accelerating corrosion. For the Cu-bearing steels 0Ni2Cu2 and 0Ni2Cu6, the thermally treated samples have ε-Cu dispersed on the surface, which results in the deactivation of SRBs upon contact due to its toxicity. Hence, no complete biofilms form in the first stage, preventing the occurrence of electron exchange between SRBs and the steel substrate and the consequent corrosion due to metabolic products. Furthermore, as corrosion advances into the second and third stages, Cu oxides deposit on the steel surface, enhancing the bactericidal nature of the experimental steel and forming an acid-resistant and dense oxide layer that effectively slows down the corrosion rate. The surface of 0Ni2Cu6 steel is sheathed with much more densely packed ε-Cu due to higher Cu content. Cu integration not only enhances antibacterial activity and increases corrosion resistance, but also promotes a fast and stable formation of an acid-resistant oxide layer, significantly augmenting the steel’s corrosion resistance.

## 4. Conclusions

The corrosion rule of three kinds of steel in an SRB environment was found: with increasing of Cu content in steel, the resistance of carbon steel is better, and the higher the Cu content, the more obvious the antibacterial property. Therefore, the corrosion products of these three kinds of steel were studied in detail, and the special corrosion mechanism of Cu steel was obtained. The details are as follows:

1. The corrosion rates of the three steels were accelerated in the presence of SRB, evidenced by an increase in weight loss of 9.75%, 6.64%, and 5.43% for the three steels, respectively. The copper-containing steels consistently exhibited lower corrosion rates than the Cu-free Q355 samples, regardless of bacterial presence. Furthermore, higher Cu content granted better corrosion resistance.
2. The copper-containing steels, 0Ni2Cu2 and 0Ni2Cu6, demonstrated pronounced antimicrobial capabilities compared to that of the Cu-free Q355 steel. The antimicrobial performance was found to be more pronounced with higher Cu content.
3. Post-corrosion, the surface of copper-containing steels was coated with a compact CuFe_2_O_4_ layer, serving as a formidable shield against invasive ions. This protective film significantly hampered ion penetration, thereby protecting the steel matrix and elevating the material’s resistance to corrosion.

## Figures and Tables

**Table 1:** Chemical compositions of materials (wt.%).

Steel	C	Si	Mn	V	Cr	Ni	Cu	Fe
Q355	0.24	0.55	1.6	--	0.30	0.30	--	Blance
0Ni2Cu2	0.025	0.19	0.95	0.025	0.47	2.69	2.31	Blance
0Ni2Cu6	0.036	0.20	0.97	0.025	0.48	2.70	6.01	Blance

**Table 2:** Chemical compositions of seawater (g/L).

NaCl	MgSO_4_	MgCl_2_	CaCl_2_	KCl	NaHCO_3_	NaBr
26.518	3.305	2.447	1.141	0.725	0.202	0.083

**Table 3:** Chemical composition of the medium solution (g/L).

K_2_HPO_4_	NH_4_Cl	Na_2_SO_4_	Sodium Lactate	Yeast Extract	CaCl_2_	MgSO_4_	Ascorbic Acid	Ammonium Ferrous Sulfate
0.5	1.0	0.5	3.0	1.0	0.1	2.0	0.1	0.2

**Table 4:** XPS results of corrosion products of three specimens corroded for 28 days.

Atomic/%	Q355	0Ni2Cu2	0Ni2Cu6
FeOOH	1.85	2.15	1.63
Fe_2_O_3_	2.43	2.42	2.37
FeS_2_	1.98	--	--
FeS	1.98	2.71	4.95
Cu^2+^	--	0.04	0.09
Cu^+^	--	0.03	0.06

**Table 5:** Fitted data for different samples immersed in SRB for different numbers of days.

Duration	Steel No.	R_s_, Ω∙cm^2^	C_f_, Ω^−1^∙cm^−2^	R_f_, Ω∙cm^2^	C_dl_, Ω^−1^∙cm^−2^	R_ct_, Ω^−1^∙cm^−2^
3 day	Q355	1.08	----	---	6.109 × 10^−4^	4.038 × 10^2^
	0Ni2Cu2	8.822	---	---	7.583 × 10^−1^	1.080 × 10^3^
	0Ni2Cu6	12.25	---	---	6.529 × 10^−1^	7.427 × 10^3^
7 day	Q355	8.27	3.118 × 10^−1^	4.867	7.695 × 10^−2^	3.634 × 102
	0Ni2Cu2	11.95	8.859 × 10^−3^	17.24	6.809 × 10^−1^	1.097 × 10^3^
	0Ni2Cu6	23.23	1.280 × 10^−2^	18.34	1.821 × 10^−3^	3.078 × 10^3^
14 day	Q355	12.07	4.695 × 10^−1^	5.048 × 10^2^	1	2.338 × 102
	0Ni2Cu2	6.733	8.208 × 10^−1^	4.481	9.125 × 10^−1^	4.365 × 10^3^
	0Ni2Cu6	8.263	3.144 × 10^−3^	8.768	6.465 × 10^−1^	4.980 × 10^4^
28 day	Q355	6.082	8.436 × 10^−1^	1.601 × 10^1^	4.853 × 10^−1^	4.105 × 10^2^
	0Ni2Cu2	9.125	4.787 × 10^−1^	4.314 × 10^1^	3.946 × 10^−2^	1.779 × 10^5^
	0Ni2Cu6	14.28	7.469 × 10^−1^	5.076 × 10^2^	2.529 × 10^−2^	3.411 × 10^5^

**Table 6:** Polarization fitting data for different samples immersed in SRB for different numbers of days.

Duration	Steel No.	E_corr_, V	I_corr_, A∙cm^−2^
3 day	Q355	−1.06	3.757 × 10^−4^
	0Ni2Cu2	−1.04	3.032 × 10^−4^
	0Ni2Cu6	−1.045	1.648 × 10^−5^
7 day	Q355	−0.991	1.425 × 10^−4^
	0Ni2Cu2	−1.002	2.867 × 10^−4^
	0Ni2Cu6	−1.010	1.083 × 10^−4^
14 day	Q355	−1.066	7.838 × 10^−4^
	0Ni2Cu2	−0.926	2.867 × 10^−4^
	0Ni2Cu6	−1.002	2.514 × 10^−4^
28 day	Q355	−1.131	5.556 × 10^−3^
	0Ni2Cu2	−1.088	4.584 × 10^−4^
	0Ni2Cu6	−1.058	3.905 × 10^−4^

## Data Availability

Data are contained within the article.
